# The Four-Dimensional Symptom Questionnaire (4DSQ) in the general population: scale structure, reliability, measurement invariance and normative data: a cross-sectional survey

**DOI:** 10.1186/s12955-016-0533-4

**Published:** 2016-09-15

**Authors:** Berend Terluin, Niels Smits, Evelien P. M. Brouwers, Henrica C. W. de Vet

**Affiliations:** 1 Department of General Practice and Elderly Care Medicine & EMGO Institute for Health and Care Research, VU University Medical Center, Amsterdam, The Netherlands; 2 Research Institute of Child Development and Education, University of Amsterdam, Amsterdam, The Netherlands; 3 Scientific Center for Care and Welfare (Tranzo), Tilburg University, Tilburg, The Netherlands; 4 Department of Epidemiology and Biostatistics, EMGO Institute for Health and Care Research, VU University Medical Center, Amsterdam, The Netherlands

**Keywords:** Distress, Depression, Anxiety, Somatization, Confirmatory factor analysis, Bifactor model, Measurement invariance, Differential item functioning, Normed reference data

## Abstract

**Background:**

The Four-Dimensional Symptom Questionnaire (4DSQ) is a self-report questionnaire measuring distress, depression, anxiety and somatization with separate scales. The 4DSQ has extensively been validated in clinical samples, especially from primary care settings. Information about measurement properties and normative data in the general population was lacking. In a Dutch general population sample we examined the 4DSQ scales’ structure, the scales’ reliability and measurement invariance with respect to gender, age and education, the scales’ score distributions across demographic categories, and normative data.

**Methods:**

4DSQ data were collected in a representative Dutch Internet panel. Confirmatory factor analysis was used to examine the scales’ structure. Reliability was examined by Cronbach’s alpha, and coefficients omega-total and omega-hierarchical. Differential item functioning (DIF) analysis was used to evaluate measurement invariance across gender, age and education.

**Results:**

The total response rate was 82.4 % (*n* = 5273/6399). The depression scale proved to be unidimensional. The other scales were best represented as bifactor models consisting of a large general factor and one or more smaller specific factors. The general factors accounted for more than 95 % of the reliable variance of the scales. Reliability was high (≥0.85) by all estimates. The distress-, depression- and anxiety scales were invariant across gender, age and education. The somatization scale demonstrated some lack of measurement invariance as a result of decreased thresholds for some of the items in young people (16–24 years) and increased thresholds in elderly people (65+ years). The somatization scale was invariant regarding gender and education. The 4DSQ scores varied significantly across demographic categories, but the explained variance was small (<6 %). Normative data were generated for gender and age categories. Approximately 17 % of the participants scored above average on de distress scale, whereas 12 % scored above average on de somatization scale. Percentages of people scoring high enough on depression or anxiety as to suspect the presence of depressive or anxiety disorder were 4.1 and 2.5 respectively.

**Conclusions:**

Evidence supports reliability and measurement invariance of the 4DSQ in the general Dutch population. The normative data provided in this study can be used to compare a subject’s 4DSQ scores with a general population reference group.

**Electronic supplementary material:**

The online version of this article (doi:10.1186/s12955-016-0533-4) contains supplementary material, which is available to authorized users.

## Background

The Four-Dimensional Symptom Questionnaire (4DSQ) is a self-report questionnaire comprising four scales measuring distress, depression, anxiety and somatization [1]. The 4DSQ was developed in Dutch general practice and is currently used by increasingly larger numbers of family and occupational physicians, physiotherapists, social workers, counsellors, and primary care psychologists. The 4DSQ is intended to be used in both clinical and research settings. The distress scale aims to measure the kind of symptoms people experience when they are “under stress” as a result of high demands, psychosocial difficulties, daily hassles, life events, or traumatic experiences [2]. The distress scale measures people’s most general, most basic response to stress of any kind. The distress score reflects any mental health problem and indicates the degree of subjective psychological suffering [3]. The depression scale measures symptoms that are relatively specific to depressive disorder, notably, anhedonia and negative cognitions [4, 5]. The anxiety scale measures symptoms that are relatively specific to anxiety disorder [6]. Scores on the 4DSQ depression and anxiety scales indicate the likelihood of a (DSM-IV) depressive or anxiety disorder [7, 8]. The somatization scale measures symptoms of somatic distress and somatoform disorder [9, 10].

The 4DSQ has been validated in selected, mainly clinical samples from primary care settings [1, 7, 8, 11]. The present paper aims to evaluate the 4DSQ scales’ measurement properties in the general Dutch population and to provide normative data. In particular, we examined the following scale characteristics:

- the scales’ factor structures,
- the scales’ reliability,
- the scales’ measurement invariance with respect to gender, age and education,
- the scales’ score distributions across demographic categories,
- normative data for the general Dutch population.

## Methods

### Design and participants

The present study was performed in the LISS panel (LISS: Longitudinal Internet Study in the Social Sciences), an Internet panel consisting of a representative sample of Dutch-speaking non-institutionalized individuals from approximately 5,000 households in the Netherlands, managed by CentERdata [12]. The LISS panel is based on a true probability sample drawn from the population register by Statistics Netherlands. All eligible people were approached in traditional ways (i.e., by letters, telephone calls and/or house visits) with an invitation to participate in the panel. Households that could not otherwise participate were provided with a computer and Internet connection. As participation was not open for people not included in the sample drawn by Statistics Netherlands, self-selection is not an issue in the LISS panel. Imminent under-coverage of specific groups (e.g., youths, ethnic minorities) due to reduced willingness to participate or increased attrition is actively counteracted by targeted oversampling of those groups in additional “refreshment” samples [12]. Panel members complete online questionnaires on a monthly basis receiving a reimbursement of €7.50 for a questionnaire of 30 min. In July 2013, the 4DSQ was presented to a random sample (*n* = 771) of all available panel members aged 16 years and older. In October 2013, the 4DSQ was presented to all then available panel members of 16 years and older, except those who had already completed the 4DSQ in July (the October questionnaire was presented to 5659 participants). For the present study the July and October samples were pooled.^1^

### Measurements

The 4DSQ comprises four symptom scales: distress (16 items), depression (6 items), anxiety (12 items), and somatization (16 items). The 4DSQ uses a time-frame reference of 7 days. The items are answered on a 5-point frequency scale from “no” to “very often or constantly”. In order to calculate sum scores the responses are coded on a 3-point scale: “no” (0 points), “sometimes” (1 point), “regularly”, “often”, and “very often or constantly” (2 points). By lumping the response categories “regularly”, “often”, and “very often or constantly” together relatively more weight is put on the number of symptoms experienced than on their perceived frequency. The 4DSQ is freely available for non-commercial use at www.4dsq.eu.

### Analyses

#### Weighting

In order to account for selective non-response (of e.g. people with low income) and to obtain results applicable to the general Dutch population, the responders were weighted using inverse response probability weighting [13]. All analyses were performed on weighted data.

#### Confirmatory factor analysis

The total sample of responders was randomly divided into two equally sized groups, a “training set” (*n* = 2636) that was used for model selection, and a “validation set” (*n* = 2637) that was used for validation of the models obtained in the training set [14].

We examined the latent structure of the 4DSQ scales using scale wise confirmatory factor analyses (CFA), using the package “lavaan” version 0.5-17 in R 3.1.2 [15, 16]. The item responses were treated as ordered categories. Diagonally weighted least squares (DWLS) was used for model estimation [17] and mean and variance adjusted test statistics were computed. Fit measures indicating good fit included the comparative fit index (CFI) >0.95, Tucker-Lewis index (TLI) >0.95 and root mean square error of approximation (RMSEA) <0.06 [18]. An RMSEA value <0.05 indicates “close fit” to the data [19]. In addition, we examined the matrix of residual correlations and aimed for less than 5 % of the residual correlations (in absolute values) greater than 0.1. For each scale, we started by fitting a one-factor model in the training set. Informed by the modification indices, improved model fit was iteratively accomplished by allowing residual item variance to correlate (but only when the items shared specific content justifying correlated residual variance). Note that correlated item residuals suggest the presence of additional “specific” factors beyond the general factor of the scale [20]. Therefore, a fitting one-factor model with correlated residual variances was transformed into a corresponding bifactor model by defining the items with correlated residuals as indicators of one or more “group” (or specific) factors [21]. The bifactor model is characterized by one large general factor on which all items are loading, and one or more smaller group factors on which subsets of items load [22]. Psychological constructs are often “multifaceted” and the bifactor model allows to model a general factor representing the overall target construct of the scale, whereas one or more group factors model specific “facets” of the construct [23]. The bifactor models obtained in the training set were subsequently validated in the validation set using the model parameters (factor structure and loadings) from the training set.

To provide insight into the relationships between the (sub)scales we obtained factor scores for the general and specific factors in the validation set, and calculated Pearson product moment correlations.

#### Reliability

Reliability was assessed in the total sample. Conventional Cronbach’s alpha values were calculated using the R-package “psych” [24]. Cronbach’s alpha represents a lower bound to reliability [25]. In addition, we calculated coefficients omega-total and omega-hierarchical based on the standardized factor loadings derived from the bifactor models obtained in the CFAs, as described by Reise [22]. Omega-total reflects the proportion of the total variance that is due to all common (general and group) factors, whereas omega-hierarchical reflects the proportion of the total variance that is accounted for by the general factor alone [21]. Omega-hierarchical can be viewed as reflecting the general factor saturation of a scale [26].

In addition to reliability, we calculated standard errors of measurement (SEM) using the formula *SEM* = *SD* * √(1 – *r*), in which *SD* is the standard deviation and *r* is the reliability of the scale. We used omega-total for *r*. SEM is a useful measure of measurement precision.

#### Measurement invariance

Measurement invariance is present when a scale measures the same construct (e.g., distress) in the same way across different groups of responders (e.g., women and men) [27]. Then the scale scores can be assumed to convey the same meaning (i.e., validity) across those groups. Psychological constructs, such as distress, are often measured using multi-item questionnaires. The responses to the items are thought to be driven by the latent (i.e., not directly observable) construct – or trait (e.g., distress). Thus, the items’ responses are indicators of the underlying latent trait and together provide information about responders’ positions on the trait. The relationship between item responses and the underlying trait is defined by two characteristics, the correlation between the trait and the item responses, and the “threshold” of the item relative to the trait. The threshold of the item is represented by the level of the latent trait at which 50 % of the respondents endorse the item. Items are said to “function the same” when they have the same item characteristics (i.e., correlation and threshold) with respect to the underlying trait. When the items of a scale function the same in different groups, the scale can be assumed to have the same validity in these groups. Whether or not items function the same in different groups can be assessed using “differential item functioning” (DIF) analysis [28]. There are several methods to detect DIF, but no single method has proven superiority over the other methods [29]. Some authors, therefore, suggest using two different methods [30]. We used a parametric method, hybrid ordinal logistic regression (HOLR) as implemented in the R-package “lordif” version 0.2.2 [31], and a non-parametric method, the Mantel-Haenszel (M-H) method as implemented in the statistical program jMetrik 3.0 [32]. We tested DIF with respect to gender, age (age groups: 15–24, 25–44, 45–64 and 65+ years) and education (categorized in lower, intermediate, and higher education) in the training set. The criterion for DIF was the group factor explaining >2 % of the item variance (McFadden’s R^2^) in the HOLR-method, or a standardized mean difference (SMD) in item score >0.1 between groups in the M-H-method. Unlike the M-H-method, the HOLR-method is capable of testing more than 2 groups simultaneously. Using the M-H-method, we tested any pair of groups at the time (e.g., lower education versus intermediate education, lower education versus higher education, and intermediate education versus higher education). To account for multiple testing we adopted *p* < 0.001 as significance level.

The effect of DIF on the mean scale score (i.e., differential test functioning; DTF) was subsequently evaluated in the validation set. We regressed the raw scale score on the group variable while adjusting for the sum score of the items that were found to be free of DIF in both methods. The resulting difference in mean total score between 2 groups is denoted as DTFR statistic [33]. We calculated effect sizes, denoted *d*_DTF_, by dividing the DTFR values by the scale’s standard deviation. These effect sizes can be interpreted in the usual way: 0.2 represents a small effect, 0.5 a moderate effect, and 0.8 a large effect [34].

#### Association with demographic characteristics and normative data

We examined the associations between 4DSQ scores and demographic characteristics using univariate analysis of variance (ANOVA) in the total sample.

Furthermore, we calculated normative data by gender and age group, providing the distribution parameters mean, standard deviation and skewness, and percentile scores.

## Results

### Demographics and response

In total, either in July or October 2013, the 4DSQ was presented to 6399 LISS participants (31 non-responders in July received the 4DSQ again in October). The response rate was 5273/6399 (82.4 %). The demographic characteristics of the total sample and the responders are presented in Table 1. Standardized residuals >2 or < −2 indicate over- or underrepresentation among the responders. Underrepresented were younger and unmarried people, people with paid work or studying/school going, people with low personal income, and people with a non-Western or unknown ethnicity. Overrepresented were retired and widowed people. After weighting, the responders sample mirrored the total sample almost perfectly. There were no significant differences between the responders in July and the responders in October, expect for age: the July responders were on average 1.7 years older than the October responders (see Additional file 1). This probably reflected differences between the panel members available in July and those available in October. There were no significant differences between the training set and the validation set of responders (see Additional file 2).

**Table 1:** Demographic characteristics of the response groups

Characteristic	Responders (*n* = 5273)	Non-responders (*n* = 1126)	Effect sizes^a^/*p*-values^b^	Weighted responders (*n* = 5273)	Total group (*n* = 6399)
Age (mean, sd)	50.9 (17.6)	39.3 (16.2)	*d* = 0.67 *p* <0.001	48.8 (17.9)	48.8 (17.9)
Gender (%)			*p* 0.538		
Female	54.0	52.9	SR 0.65	53.9	53.8
Male	46.0	47.1	SR −0.65	46.1	46.2
Ethnicity (%)			*p* <0.001		
Native Dutch	84.6	75.8	SR 7.14	83.3	83.0
Foreign, Western country	7.0	7.0	SR −0.07	7.0	7.0
Foreign, Non-Western country	4.4	6.7	SR −3.22	4.8	4.8
Unknown	4.1	10.6	SR −8.89	4.9	5.2
Education (%)			*p* <0.001		
Primary	9.0	12.1	SR −3.18	9.5	9.5
Lower vocational	25.1	18.7	SR 4.62	24.0	24.0
Secondary	11.1	15.1	SR −3.80	11.8	11.8
Middle vocational	23.0	23.6	SR −0.46	23.1	23.1
Higher vocational	22.8	20.8	SR 1.47	22.4	22.4
University	8.7	9.6	SR −0.91	8.9	8.9
Unknown	0.3	0.2	SR 0.54	0.3	0.3
Marital status (%)			*p* <0.001		
Married	57.5	42.2	SR 9.37	54.8	54.8
Divorced	9.1	9.2	SR −0.12	9.1	9.1
Widowed	5.4	2.9	SR 3.47	5.0	5.0
Never married	28.0	45.6	SR −11.63	31.1	31.1
Employment status (%)			*p* <0.001		
Paid work	49.0	58.9	SR −5.99	50.8	50.8
Unemployed	3.5	2.8	SR 1.30	3.4	3.4
Disabled	3.7	4.4	SR −0.98	3.8	3.8
School or study	8.9	19.6	SR −10.49	10.8	10.8
Retired	22.4	6.0	SR 12.58	19.5	19.5
Household	8.7	5.2	SR 3.83	8.1	8.1
Other	3.7	3.1	SR 0.94	3.6	3.6
Monthly net income (%)			*p* <0.001		
0–500 Euro	17.0	23.0	SR −4.71	18.2	18.1
501–1500 Euro	35.9	31.3	SR 2.98	35.1	35.1
1501–2500 Euro	31.7	28.0	SR 2.46	31.2	31.1
> 2500 Euro	9.9	10.3	SR −0.43	10.0	10.0
Unknown	5.4	7.5	SR −2.66	5.6	5.8

^a^ *d*: Cohen’s delta in case of continuous variables, *SR* standardized residuals in case of categorical variables

^b^ *t*-test in case of continuous variables; Chi-square test in case of categorical variables

### Confirmatory factor analysis

#### Distress

**Fig. 1:**
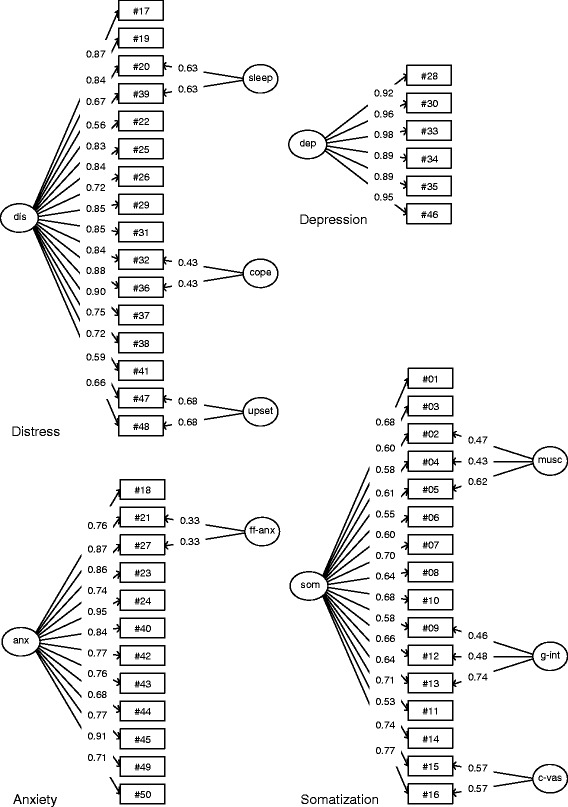
Latent structure models of the 4DSQ scales. The depression model is strictly unidimensional. The other scales demonstrate bifactor structures. Boxes represent items and circles latent factors. The general factors are represented by “dis”, “dep”, “anx”, and “som”. The other factors represent group factors: “sleep” = disturbed sleep, “cope” = failure to cope, “upset” = symptoms related to past upsetting events, “ff-anx” = free floating anxiety, “musc” = musculoskeletal symptoms, “g-int” = gastro-intestinal symptoms, “c-vas” = cardiovascular symptoms. Coefficients are standardized factor loadings

The one-factor model of the distress scale with correlated residuals in 3 item doublets demonstrated good fit to the data in the training set (Table 2). No more than 4 residual correlations (3.3 %) exceeded 0.10 (in absolute values); none of the residuals exceeded 0.20. The correlated item doublets were, in order of importance, #47 – #48, referring to consequences of upsetting events, #20 – #39, related to disturbed sleep, and #32 – #36, expressing failure to cope. The corresponding bifactor model fitted the data well. In order to allow identification of the model, the loadings of the item doublets were constrained to be equal. The same bifactor model in the validation set, using the factor loadings from the training set, fitted the data slightly better. The confidence interval of the RMSEA, lying entirely below 0.05, indicated close fit of the model to the data. Figure 1 displays the bifactor model of distress in the upper left part.

**Table 2:** Confirmatory factor analysis: fit indices of the 4DSQ scales

4DSQ scale	Model	Chi-square	df	*p*-value	CFI	TLI	RMSEA	90 % CI of RMSEA
Distress	One-factor^a^	2819.06	104	0.000	0.987	0.975	0.100	0.096 – 0.103
	One-factor with correlated residuals^a^	763.11	101	0.000	0.994	0.993	0.051	0.047 – 0.053
	Bifactor^a^	760.35	101	0.000	0.994	0.993	0.051	0.046 – 0.053
	Bifactor^b^	765.19	116	0.000	0.996	0.996	0.045	0.043 – 0.049
Depression	One-factor^a^	53.49	9	0.000	0.999	0.998	0.046	0.032 – 0.054
	One-factor^b^	72.96	14	0.000	0.999	0.999	0.040	0.031 – 0.049
Anxiety	One-factor^a^	147.69	54	0.000	0.996	0.995	0.028	0.020 – 0.030
	One-factor with correlated residuals^a^	121.65	53	0.000	0.997	0.996	0.025	0.017 – 0.027
	Bifactor^a^	121.28	53	0.000	0.997	0.996	0.025	0.017 – 0.027
	Bifactor^b^	283.45	64	0.000	0.995	0.995	0.035	0.031 – 0.040
Somatization	One-factor^a^	1529.21	104	0.000	0.954	0.947	0.073	0.069 – 0.075
	One-factor with correlated residuals^a^	370.12	97	0.000	0.991	0.988	0.034	0.029 – 0.036
	Bifactor^a^	375.02	97	0.000	0.991	0.988	0.034	0.029 – 0.036
	Bifactor^b^	535.96	116	0.000	0.989	0.989	0.036	0.034 – 0.040

*CFI* comparative fit index

*TLI* Tucker-Lewis index

*RMSEA* root mean square error of approximation

*CI* confidence interval

^a^training set

^b^validation set

#### Depression

The one-factor model of the depression scale demonstrated good fit without the need to allow residuals to correlate (Table 2). Consequently, there was no need to define group factors in a bifactor model. The one-factor model was replicated in the validation set, demonstrating close fit to the data. The one-factor model of depression is shown in the upper right part of Fig. 1.

#### Anxiety

The one-factor model of the anxiety scale, with one residual correlation (between #21 and #27, both items refer to free floating anxiety), demonstrated good fit (Table 2). The corresponding bifactor model was confirmed in the validation set, showing close fit to the data. The model is shown in the lower left part of Fig. 1.

#### Somatization

The one-factor model of the somatization scale needed correlated residuals between two item triplets and one item doublet to obtain good fit (Table 2). The item triplets were: #09 – #12 – #13 (gastro-intestinal symptoms) and #02 – #04 – #05 (musculoskeletal symptoms), whereas the item doublet concerned items #15 – #16 (cardiovascular or thoracic symptoms). The corresponding bifactor model fitted well in the training set. This model was replicated in the validation set, showing close fit to the data (Table 2). The model is displayed in the lower right part of Fig. 1.

#### Correlations between factors

Table 3 displays the correlation matrix of the 4DSQ factor scores. The correlations between the general factors were largely in agreement with correlations between the raw scale scores in previous studies [1]. The correlations with the (residualized) specific factors were all small.

**Table 3:** Pearson product moment correlations between the factor scores of the 4DSQ general and specific factors

Factor		1	2	3	4	5	6	7	8	9	10	11
Distress^a^	1	1										
Upsetting events	2	0.050	1									
Sleep	3	0.074	0.032	1								
Coping	4	0.095	−0.076	−0.091	1							
Depression^a^	5	**0.704** ^a^	−0.007	−0.029	0.220	1						
Anxiety^a^	6	**0.679** ^a^	0.123	0.061	0.052	**0.584** ^a^	1					
Free floating anxiety	7	0.173	−0.005	0.053	−0.056	0.116	0.134	1				
Somatization^a^	8	**0.655** ^a^	0.038	0.155	0.021	**0.454** ^a^	**0.567** ^a^	0.083	1			
Cardio-vascular	9	0.055	0.018	0.018	−0.006	0.042	0.054	0.038	0.148	1		
Gastro-intestinal	10	0.131	−0.004	0.008	−0.020	0.069	0.122	0.025	0.118	−0.115	1	
Musculoskeletal	11	0.149	0.011	0.030	−0.005	0.046	0.055	−0.024	0.202	−0.125	−0.110	1

^a^General factors; bold correlations are correlations between the 4DSQ scales’ general factors

### Reliability

The different reliability coefficients are summarized in Table 4. All coefficients were over 0.85 and many were over 0.90, suggesting (more than) adequate reliability of the scales. Given the omega-hierarchical values, the general factors accounted for the lion’s share of the scales’ total reliable variance. The SEM values were relatively small compared with the scales’ ranges. For instance, the SEM of the distress scale (range 32 points) was 1 point, indicating that the 95 % confidence interval of an observed distress score of *x* was *x*–1.96 to *x* + 1.96.

**Table 4:** Reliability coefficients and standard errors of measurement (SEM) of the 4DSQ scales

4DSQ scale	Reliability coefficients			SEM
	Cronbach’s alpha	Omega-total	Omega-hierarchical	
Distress	0.926	0.976	0.952	1.00
Depression	0.909	0.976	0.976	0.30
Anxiety	0.879	0.963	0.959	0.53
Somatization	0.845	0.944	0.896	1.17

### Measurement invariance

**Fig. 2:**
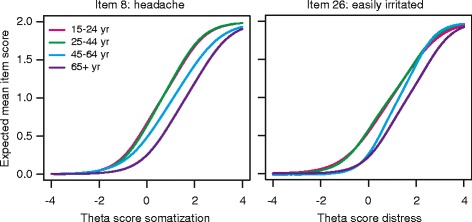
Illustration of differential item functioning (DIF) by age. Expected mean item scores as a function of the latent trait score derived from item response theory (IRT) modelling, accounting for DIF. The left-hand panel displays the mean item score of item 8 as a function of the trait score for somatization, by age category. The right-hand panel displays the mean item score of item 26 as a function of the trait score for distress, by age category. The graphs were obtained from the program “lordif”

Items that demonstrated DIF for gender, age or education in the training set are listed in Table 5. The items of the depression scale were all free of DIF. Regarding the other scales, a total of 17 items were found to have DIF by either method (i.e., HOLR or M-H). Only 4 items were flagged for DIF by both methods. Most DIF was due to the factor age. Figure 2 illustrates DIF by age for two items, showing the expected item score as a function of the trait score, i.e., the DIF-free item response theory theta score. The slope of the curves represent the item-trait correlation. The horizontal shift of the curves for different age groups indicate different item thresholds across the age groups. The thresholds for headache (left panel) and irritability (right panel) increased progressively with increasing age. Older people reported less headache and irritability than younger people at comparable levels of somatization and distress respectively.

**Table 5:** 4DSQ items identified with differential item functioning (DIF)

4DSQ scale	Item	Item description	DIF^a^	Effect size^b^	Direction^c^
Distress	#25	Feeling tense	Age	SMD = 0.10/0.14	16-44 years > 65+ years
	#26	Feeling easily irritated	Age	R^2^ = 3.58; SMD = 0.17/0.25	16-44 years > 45+ years
	#39	Difficulty getting to sleep	Age	SMD = 0.13	16-24 years > 25–44 years
			Education	SMD = 0.11	lower education > higher education
	#47	Fleeting images of past upsetting events	Age	R^2^ = 2.45; SMD = −0.16/-0.17	16-44 years < 65+ years
	#48	Put aside thoughts about past upsetting events	Age	SMD = −0.11/-0.14	16-44 years < 65+ years
Anxiety	#43	Afraid of public transport	Age	R^2^ = 3.70	25-44 years > 45+ years; 25–44 years > 16–24 years
	#44	Afraid of embarrassment with other people	Age	R^2^ = 2.63; SMD = 0.15	16-24 years > 45+ years
Somatization	#1	Dizziness/light-headedness	Age	SMD = 0.15/0.18	16-24 years > 45+ years
	#2	Painful muscles	Age	SMD = −0.12/-0.16	16-44 years < 65+ years
			Education	SMD = 0.12	lower education > higher education
	#6	Excessive sweating	Age	SMD = 0.11	45-64 years > 65+ years
	#8	Headache	Age	R^2^ = 3.93; SMD = 0.17/0.42	16-44 years > 45–64 years > 65+ years
			Gender	SMD = 0.13	Female > male
	#9	Bloated feeling in abdomen	Age	SMD = 0.13	16-24 years > 65+ years
	#10	Blurred vision	Age	SMD = −0.12	25-44 years < 65+ years
	#12	Nausea or upset stomach	Age	SMD = 0.18/0.22	16-24 years > 45+ years
	#13	Pain in abdomen or stomach	Age	SMD = 0.18/0.19	16-24 years > 45+ years
	#14	Tingling in fingers	Age	SMD = −0.11	25-44 years > 65+ years
	#16	Pain in the chest	Gender	R^2^ = 3.05	Female < male

^a^DIF: group factor associated with differential item functioning

^b^Effect size: R^2^: item score variance (%) explained by the group factor (hybrid ordinal logistic regression method); *SMD* standardized mean difference (Mantel-Heanszel method; multiple SMDs are noted as a range, e.g. 0.10/0.14 means from 0.10 to 0.14)

^c^Direction of DIF: one group tends to score higher (>) or lower (<) than the other group due to DIF

Differential test functioning (DTF; i.e., the effect of DIF on the scale score) is presented in Table 6. The largest DTF effect concerned the effect of age on the somatization score: younger people (16–24 years) scored on average 1.234 scale points higher on the somatization scale than elderly people (65+ years), adjusted for the true level of somatization. Similarly, they scored on average 1.234 – 0.561 = 0.673 scale points higher than young adults (25–44 years) and 1.234 – 0.355 = 0.879 scale point higher than older adults (45–64 years), all adjusted for differences in somatization trait levels across the age groups. This DTF effect resulted from some of the somatization items having lower thresholds in younger people (16–24 years) than in older people and some (partly other) somatization items having higher thresholds in elderly people (65+ years) than in younger people. In terms of effect size, however, the DTF effect of age on the somatization score constituted only a small effect, and only when comparing the youngest group (16–24 years) with the oldest group (65+ years). All other DTF effects were negligible from a practical point of view (i.e., considering the effect sizes *d*_DFT_).

**Table 6:** Differential test functioning (DTF) of the 4DSQ scales

4DSQ scale	Factor	DTFR	95 % CI	*p*	*d*_DTF_	95 % CI
Distress	Education^a^					
	- Lower	0.127	0.073; 0.180	0.000	0.019	0.011; 0.027
	- Intermediate	0.044	−0.010; 0.099	0.107	0.007	−0.001; 0.015
	Age^b^					
	- 16–24 years	0.287	0.105; 0.469	0.002	0.043	0.016; 0.070
	- 25–44 years	0.066	−0.080; 0.212	0.377	0.010	−0.012; 0.031
	- 45–64 years	0.095	−0.043; 0.233	0.175	0.014	−0.006; 0.035
Anxiety	Age^b^					
	- 16–24 years	0.013	−0.050; 0.075	0.686	0.004	−0.017; 0.026
	- 25–44 years	−0.063	−0.113; −0.013	0.014	−0.022	−0.039; −0.004
	- 45–64 years	−0.048	−0.096; −0.001	0.046	−0.016	−0.033; −0.000
Somatization	Education^a^					
	- Lower	0.083	0.020; 0.146	0.010	0.016	0.004; 0.029
	-Intermediate	−0.013	−0.077; 0.050	0.680	−0.003	−0.015; 0.010
	Age^b^					
	- 16–24 years	1.234	0.930; 1.539	0.000	0.245	0.185; 0.305
	- 25–44 years	0.561	0.316; 0.806	0.000	0.111	0.063; 0.160
	- 45–64 years	0.355	0.123; 0.587	0.003	0.070	0.024; 0.116
	Gender^c^	−0.114	−0.163; −0.064	0.000	−0.023	−0.032; −0.013

DTFR: effect of differential test functioning (DTF; i.e., the effect of differential item functioning on the mean scale score)

*d*_DTF_: effect size of differential test functioning (DTFR / standard deviation of the scale score)

^a^reference: higher education

^b^reference: 65+ years

^c^reference: female

### Associations with demographic characteristics

Table 7 demonstrates that the mean 4DSQ scores for distress, depression, anxiety and somatization varied significantly across demographic characteristics. Women scored higher than men (with the exception of depression; *p* = 0.054). Younger people (16–24 years) scored higher and elderly people (65+ years) scored lower than “working age” people (25–64 years). People of non-Dutch descent scored higher than native Dutch people. People with lower education scored higher than people with higher education. Divorced people scored higher than married people. Disabled and unemployed people scored higher than people with paid work. And, finally, there was a clear (negative) gradient of the 4DSQ scores with the personal income level. Nevertheless, the explained variance, expressed as Eta-squared, did not exceed 6 % for any of the characteristics explaining any of the 4DSQ scores. The largest effects were observed for somatization, 5.6 % of its variance being explained by employment status. Employment status was the demographic characteristic with the largest effects on all 4DSQ scores, explaining 4.4 % of distress, 3.2 % of depression, 4.0 % of anxiety, and 5.6 % of somatization.

**Table 7:** Association of 4DSQ scores with demographic characteristics

Characteristic	Distress			Depression			Anxiety			Somatization		
	Mean (sd)	F*	Eta^2^	Mean (sd)	F*	Eta^2^	Mean (sd)	F*	Eta^2^	Mean (sd)	F*	Eta^2^
Gender		107.88	0.020		3.71*	0.001		44.95	0.008		182.61	0.033
Female	6.45 (6.82)			0.76 (2.04)			1.36 (3.00)			5.80 (5.23)		
Male	4.61 (5.91)			0.66 (1.82)			0.86 (2.37)			3.98 (4.39)		
Age groups		13.78	0.008		5.71	0.003		11.82	0.007		11.59	0.007
16-24 years	6.76 (7.10)			0.95 (2.29)			1.67 (3.39)			5.92 (5.32)		
25-44 years	5.71 (6.80)			0.70 (1.88)			1.13 (2.90)			4.87 (4.99)		
45-64 years	5.61 (6.45)			0.74 (2.01)			1.06 (2.68)			4.94 (4.95)		
65+ years	4.78 (5.59)			0.57 (1.67)			0.92 (2.09)			4.55 (4.56)		
Ethnicity		16.24	0.006		27.69	0.011		27.36	0.011		15.29	0.006
Native Dutch	5.32 (6.20)			0.63 (1.79)			1.00 (2.47)			4.75 (4.73)		
Foreign, Western	6.47 (7.27)			1.03 (2.47)			1.39 (3.06)			5.48 (5.14)		
Foreign, Non-Western	7.32 (8.11)			1.44 (2.73)			2.21 (4.66)			6.31 (6.70)		
Education		7.20	0.007		7.22	0.007		9.99	0.009		20.22	0.019
Primary	6.69 (7.03)			0.99 (2.32)			1.70 (3.22)			6.73 (5.82)		
Lower vocational	5.61 (6.59)			0.80 (2.09)			1.24 (2.86)			5.08 (5.25)		
Secondary	6.20 (6.92)			0.84 (2.08)			1.33 (3.12)			5.08 (4.80)		
Middle vocational	5.68 (6.57)			0.75 (2.00)			1.11 (2.75)			5.00 (4.95)		
Higher vocational	5.05 (5.95)			0.47 (1.47)			0.80 (2.16)			4.27 (4.30)		
University	4.77 (5.80)			0.60 (1.76)			0.84 (2.42)			4.23 (4.24)		
Marital status		32.50	0.018		24.96	0.014		13.01	0.007		10.98	0.006
Married	4.85 (5.91)			0.52 (1.65)			0.94 (2.51)			4.67 (4.79)		
Divorced	7.36 (7.54)			1.22 (2.52)			1.54 (3.10)			5.81 (5.60)		
Widowed	5.95 (5.80)			0.85 (1.92)			0.93 (1.82)			4.56 (4.63)		
Never married	6.34 (7.00)			0.89 (2.18)			1.37 (3.08)			5.29 (5.00)		
Employment status		40.42	0.044		28.80	0.032		36.45	0.040		52.12	0.056
Paid work	5.10 (6.13)			0.57 (1.69)			0.85 (2.32)			4.42 (4.46)		
Unemployed	7.14 (7.33)			1.40 (2.76)			1.40 (3.19)			5.41 (5.80)		
Disabled	11.23 (8.92)			2.16 (3.30)			3.31 (5.10)			10.03 (6.59)		
School or study	6.77 (6.92)			0.93 (2.24)			1.67 (3.25)			5.87 (5.15)		
Retired	4.53 (5.25)			0.53 (1.56)			0.83 (1.94)			4.35 (4.48)		
Household	5.88 (6.54)			0.70 (1.96)			1.35 (3.07)			5.54 (5.37)		
Other	6.81 (7.89)			1.00 (2.49)			1.92 (3.77)			6.03 (5.48)		
Monthly net income		43.58	0.026		22.37	0.013		39.66	0.023		64.57	0.039
0–500 Euro	6.47 (7.05)			0.93 (2.29)			1.63 (3.45)			5.97 (5.48)		
501–1500 Euro	6.48 (6.93)			0.88 (2.17)			1.38 (2.94)			5.75 (5.33)		
1501–2500 Euro	4.68 (5.64)			0.52 (1.56)			0.72 (2.02)			4.00 (4.03)		
> 2500 Euro	3.77 (4.91)			0.31 (1.02)			0.49 (1.73)			3.54 (3.99)		

*all F-values: *p* < 0.001, except for depression*gender: F(1,5271) = 3.71, *p* = 0.054

It is important to note that DTF was responsible for most of the differences in mean somatization scores across the age categories. Taking DTF into account (and taking the age group 65+ as reference), the youngest group (16–24 years) scored 5.92 – 1.23 = 4.69 for somatization, which is only marginally higher than the mean somatization score of the oldest group (65+ years): 4.55. Similarly, young adults (25–44 years) scored 4.87 – 0.56 = 4.31 and older adults (45–64 years) scored 4.94 – 0 36 = 4.58 on somatization after taking DTF into account. DTF did not account for other differences in 4DSQ scores.

### Normative data by gender and age

Table 8 provides normative data by gender and age category. Clearly, the 4DSQ scores were positively skewed, as is normally the case with symptom questionnaires in non-clinical populations [35]. The depression and anxiety scores were more heavily skewed than the distress and somatization scores as a result of sizeable “floor effects”: 77.8 % of all women and 79.7 % of all men scored zero on the depression scale, and 62.9 % of the women and 73.1 % of the men scored zero on the anxiety scale. In contrast, only 16.4 % of the women and 25.6 % of the men scored zero on the distress scale, and 12.5 % of the women and 21.5 % of the men scored zero on the somatization scale.

**Table 8:** Normative 4DSQ data from the Dutch general population, by gender and age

	Women				Men			
	16–24 years	25–44 years	45–64 years	65+ years	16–24 years	25–44 years	45–64 years	65+ years
	*n* = 411	*n* = 804	*n* = 1048	*n* = 578	*n* = 278	*n* = 635	*n* = 915	*n* = 606
Distress								
Mean	7.94	6.60	6.22	5.61	5.01	4.58	4.91	4.00
Standard deviation	7.65	7.27	6.46	6.00	5.79	5.98	6.37	5.05
Skewness	1.09	1.52	1.48	1.65	1.89	1.98	1.97	2.19
Minimum	0	0	0	0	0	0	0	0
Median	6	4	4	4	3	2	3	2
Maximum	32	32	32	30	30	30	32	32
Percentiles								
5	0	0	0	0	0	0	0	0
20	1	1	1	1	0	0	0	0
40	4	3	3	3	2	2	1	1
60	8	6	6	5	5	3	4	3
80	14	11	11	9	9	7	8	7
95	24	24	20	18	17	19	19	14
Depression								
Mean	1.08	0.74	0.74	0.61	0.76	0.64	0.73	0.53
Standard deviation	2.49	2.03	2.03	1.71	1.95	1.69	1.98	1.63
Skewness	2.87	3.65	3.60	3.80	3.16	3.68	3.59	4.51
Minimum	0	0	0	0	0	0	0	0
Median	0	0	0	0	0	0	0	0
Maximum	12	12	12	12	12	12	12	12
Percentiles								
5	0	0	0	0	0	0	0	0
20	0	0	0	0	0	0	0	0
40	0	0	0	0	0	0	0	0
60	0	0	0	0	0	0	0	0
80	1	1	1	1	1	1	1	0
95	7	5	5	5	6	4	5	3
Anxiety								
Mean	2.13	1.35	1.19	1.14	1.01	0.84	0.91	0.72
Standard deviation	3.77	3.20	2.78	2.33	2.60	2.44	2.56	1.81
Skewness	2.90	4.02	3.81	3.46	4.74	4.92	4.84	5.00
Minimum	0	0	0	0	0	0	0	0
Median	0	0	0	0	0	0	0	0
Maximum	24	24	24	18	21	24	23	21
Percentiles								
5	0	0	0	0	0	0	0	0
20	0	0	0	0	0	0	0	0
40	0	0	0	0	0	0	0	0
60	1	0	0	0	0	0	0	0
80	4	2	2	2	1	1	1	1
95	11	8	6	6	5	5	5	4
Somatization								
Mean	7.19	5.79	5.60	5.19	4.05	3.72	4.18	3.94
Standard deviation	5.78	5.25	5.12	4.80	3.86	4.38	4.63	4.24
Skewness	1.17	1.42	1.35	1.41	1.42	2.25	1.85	1.88
Minimum	0	0	0	0	0	0	0	0
Median	6	5	4	4	3	3	3	3
Maximum	32	30	30	31	25	29	28	27
Percentiles								
5	0	0	0	0	0	0	0	0
20	2	1	1	1	0	0	0	1
40	5	3	3	3	2	2	2	2
60	8	6	5	5	4	3	4	4
80	11	9	9	8	7	6	7	6
95	18	17	16	15	11	12	14	12

Regarding currently applicable cut-offs of the 4DSQ (see: www.4dsq.eu), most participants (at least 75 %) scored in the “normal” ranges of the 4DSQ scales (Table 9). Regarding distress and somatization 17.5 and 12.3 % of all participants scored above “normal” (i.e., >10). Even less people scored above “normal’ for depression (>2, 9.4 %) or anxiety (>3, 9.7 %). Only 4.1 % scored high enough on depression to qualify for an immediate diagnostic assessment for depressive disorder, and no more than 2.5 % scored high enough on anxiety to qualify for an immediate diagnostic assessment for anxiety disorder.

**Table 9:** Frequencies of responders by conventional 4DSQ cut-offs

4DSQ scale	Score range	Women		Men		Total	
		*n*	%	*n*	%	*n*	%
Distress	0–10	2227	78.4	2125	87.3	4352	82.5
	11–20	449	15.8	225	9.2	674	12.8
	21–32	165	5.8	84	3.5	249	4.7
Depression	0–2	2555	89.9	2223	91.3	4778	90.6
	3–5	157	5.5	124	5.1	281	5.3
	6–12	129	4.5	87	3.6	216	4.1
Anxiety	0–3	2499	88.0	2264	93.1	4763	90.3
	4–9	253	8.9	125	5.1	378	7.2
	10–24	88	3.1	44	1.8	132	2.5
Somatization	0–10	2382	83.9	2241	92.1	4623	87.7
	11–20	406	14.3	166	6.8	572	10.8
	21–32	52	1.8	26	1.1	78	1.5

## Discussion

This study examined the 4DSQ scales’ structure, reliability and measurement invariance in the general population. In addition, the study examined the 4DSQ’s associations with demographic characteristics and provided normative data by gender and age.

### Scale structure

The depression scale proved to be an almost perfectly unidimensional scale. The other scales were best represented as bifactor structures, each consisting of a large general factor underlying all the items of the scale and one or more smaller “group” or “specific” factors underlying subsets of items. The general factor represents the target construct of the scale. The smaller group factors may represent certain specific “facets” of the construct.

The distress scale contained two substantive group factors that have been found in previous studies in clinical samples and translations of the 4DSQ [36, 37]: a sleep factor (items #20 and #39) and a factor associated with having experienced past upsetting events (items #47 and #48). The sleep factor may be explained by assuming that not everyone is equally vulnerable to sleep disturbances when distressed. The upsetting events factor is probably due to the fact that not every distressed person has experienced past stressful or traumatic events. Nevertheless, the sleep items and upsetting events items still demonstrated rather high loadings on the general distress factor, providing valuable information about the general distress level. In addition, the items provide valuable information about one possible cause of distress (past upsetting events) and one possible consequence of distress (sleep disturbance).

The distress group factor consisting of item #32 (“can’t cope anymore”) and item #36 (“can’t face it anymore”) was more likely due to over-similarity of the items. An indication for over-similarity may be found in the relatively low group factor loadings relative to the general factor loadings. The anxiety scale probably also contained a group factor due to over-similarity of the items #21 (“vague feeling of fear”) and #27 (“feeling frightened”).

The somatization scale demonstrated three group factors that have also been encountered in previous studies in clinical and population samples and translations of the 4DSQ [36–38]: a musculoskeletal factor (items #02, #04 and #05), a gastrointestinal factor (items #09, #12 and #13) and a cardiovascular (or thoracic symptoms) factor (items #15 and #16). These specific factors have also been found in other studies using other scales of physical symptoms [39]. In the 4DSQ somatization scale all items contributed substantively to the general factor, but in addition some items provided extra information about certain “facets” of the clinical picture. While experiencing various levels of “general” somatization, some people tended to report relatively more musculoskeletal symptoms while others tended to report relatively more cardiovascular or gastrointestinal symptoms. This resulted in some variation within the somatization syndrome. The somatization “facets” may even be affected differentially by internal or external stressors. For instance, in residents living near a newly constructed high-voltage power line, the rise in somatization was uniquely due to a rise in musculoskeletal and gastrointestinal symptoms [38].

### Reliability

We provided Cronbach’s alpha values to allow comparison with earlier studies and other scales. Cronbach’s alpha is often used as a measure of “internal consistency reliability” but it is usually not the best reliability estimate [25, 40]. Cronbach’s alpha often underestimates a scale’s true reliability [40]. A better alternative constitutes coefficient omega, based on a “bifactor” representation of the scale’s factor structure [22]. The 4DSQ scales proved to be highly reliable (omega-total >0.90), which enables application in clinical settings (where individual scores must be interpreted). The total scale scores predominantly represent general factor variance (i.e., distress: 0.952/0.976 = 97.5 %, anxiety: 0.959/0.963 = 99.6 %, somatization: 0.896/0.944 = 94.9 %), confirming that the 4DSQ scales were “essentially unidimensional”, the total scores mainly reflecting a single common factor [41]. The depression scale only had one (general) factor. Consequently, the 4DSQ scales can safely be used as unidimensional instruments to measure their respective constructs.

### Measurement invariance

Despite the existence of some degree of differential item functioning (DIF) in 17 items, the net effect of DIF on the mean scale score was negligible in most instances. This means that the 4DSQ scales measure the same constructs in the same way across gender, age and education. The only exception concerned the effect of (young) age on the somatization score. Because young people (16–24 years) had lower thresholds for a number of somatization symptoms (e.g., headache) they tended to score on average about 1 scale point higher than people over 25 years, compared to the true level of somatization. This has consequences for the interpretation of somatization scores in young people: a score of 11 in young people (16–24 years) corresponds with a score of 10 in older people. So, without taking DIF into account, young people’s somatization scores would overestimate their true levels of somatization. To be “fair” to young people with respect to the interpretation of their somatization scores, their age-specific cut-off points of the somatization scale should be raised by 1 point. This ensures that the cut-off points retain the same meaning across all age groups.

### Associations with demographic characteristics

By and large, the associations between the 4DSQ dimensions and demographic variables were in line with what is known about risk factors for poor mental health: higher scores were associated with female gender, younger age, lower education, lower income, being divorced, being unemployed or disabled, and being an immigrant (e.g., [42–45]) However, the net effect of the demographic variables on the 4DSQ scores, in terms of explained variance (given the Eta-squared values), was small – in most cases no more than a few per cent. Remarkably, the way the 4DSQ scores varied across the demographic categories was very similar across the 4DSQ dimensions. For instance, women scored higher than men, non-Western migrants scored higher than native Dutch people, unemployed people scored higher than employed people on all four 4DSQ scales.

### Normative data

Normative data are helpful to interpret the clinical significance of individual 4DSQ scores. The “average” person, representing at least 75 % of the general population, scored in the lower third of the scale range for distress and somatization, and not at all on the depression and anxiety scales. About one in six people (17.5 %) experienced “more than average” distress, including normal, but more severe responses to psychosocial stress, loss and adversity, as well as pathological responses such as depressive or anxiety disorder.^2^ Regarding somatization, one in eight people (12.3 %) experienced more than average somatization. This group was largely overlapping with the more than average distressed group, the percentage people experiencing either more than average distress or more than average somatization or both being 22.1 %. Thus, the experience of some distress and/or some somatization is rather common among the general population. In contrast, however, the experience of specific symptoms of depressive or anxiety disorder is relatively uncommon in the general population. The 4DSQ depression score is best at detecting moderate-to-severe DSM-IV major depressive disorder, the kind of depression that is more likely requiring a specific treatment [7]. Only 4.1 % of the people experienced depression scores high enough (i.e., >5) to suspect depressive disorder. With respect to anxiety, the 4DSQ anxiety score detects the majority of anxiety disorders, especially panic disorder, agoraphobia, social phobia, obsessive compulsive disorder and posttraumatic stress disorder [8]. Only 2.5 % of the people scored high enough on anxiety (i.e., >9) to suspect one or more anxiety disorders. These figures are largely in agreement with previous general population studies [43, 46], taking into account that some studies report 12-month prevalence instead of point-prevalence and that the 4DSQ is less effective in detecting specific phobias (such as spider- and claustrophobia).

### Practical implications

The (essentially) unidimensional structure of the 4DSQ scales supports the continued use of simple sum scores. Given the fairly homogeneous factor loadings within the scales, we do not expect any added value from weighted sum scores. Moreover, researchers and practitioners can take advantage of the availability of normed data that is expressed in conventional sum scores. High reliability and measurement precision make the 4DSQ suitable for application in clinical situations.

### Limitations and strengths

This study has a number of strengths including its large sample size (*n* > 5000), the representativeness of the sample, and the high response rate (>80 %). Moreover, because detailed demographic information was available, we were able to correct for non-response bias through inverse response probability weighting. A limitation, however, is that one can never be certain that all factors associated with non-response have been accounted for. A second limitation of the study, given that depression and other moods demonstrate (some) seasonal variation [47], is that most of the data have been collected in October. However, evidence suggests that psychological symptom levels during autumn approximate the average levels across the year. A third limitation is that equivalence of the Internet-based 4DSQ compared to the paper-and-pencil version has not been established yet. However, differences between Web-based and corresponding paper-and-pencil versions of questionnaires are usually small [48–50]. Nevertheless, this is a direction for future research.

## Conclusions

In the general Dutch population, the 4DSQ comprises four reliable, (essentially) unidimensional scales measuring distress, depression, anxiety and somatization. With the exception of measuring somatization in people aged 16–24 years, the 4DSQ scales measure their respective constructs in the same way across gender, age and educational groups. Young people tend to score higher on the somatization scale than older people, and for that we recommend to raise the somatization cut-offs by 1 point for the age group 16–24 years. We have provided normative data by gender and age to assist the interpretation of individual 4DSQ scores.

## Additional files

Additional file 1: Demographic characteristics by response month. (PDF 126 kb) Additional file 2: Demographic characteristics by data set. (PDF 126 kb)

## Abbreviations

4DSQ: Four-Dimensional Symptom Questionnaire
CFA: Confirmatory factor analyses
CFI: Comparative fit index
CI: Confidence interval
DIF: Differential item functioning
DTF: Differential test functioning
HOLR: Hybrid ordinal logistic regression
M-H: Mantel-Haenszel
RMSEA: Root mean square error of approximation
SD: Standard deviation
SEM: Standard error of measurement
TLI: Tucker-Lewis index

## Footnotes

1 In July 2013 the 4DSQ was presented to all available panel members of 16 years and older in two forms, the standard present tense form and an alternative past tense form. The purpose was to examine whether these forms produced different responses. It was the original plan to present the standard form to the larger part of the panel and the alternative form to a relatively small subsample. Unfortunately, however, the forms were swapped so that the smaller subsample was presented with the standard form and the greater subsample with the alternative form. As it was suspected that the form could have an effect on the way people respond to the 4DSQ (which was later partly confirmed [51]), it was decided to present the standard form of the 4DSQ again in October to those panel members who had not completed a standard 4DSQ in July. The present study includes the responders who completed the standard present tense form of the 4DSQ, either in July or in October.

2 The relationship between distress and depression/anxiety is characterized by a non-reciprocal hierarchy [1]: when depression or anxiety are high, distress is also high, but the reverse is not true. Distress is invariably a part of depression and anxiety, but depression and anxiety are not invariable part of distress. The explanation is twofold. First, it is hard to cope with depressive and anxiety disorders. Therefore, understandably, these disorders are associated with much distress. Second, depressive and anxiety disorders can be triggered in vulnerable people when they experience severe distress from external stressors.
