# Supplementary material for: The Four-Dimensional Symptom Questionnaire (4DSQ) in the general population: scale structure, reliability, measurement invariance and normative data: a cross-sectional survey
Source: Health Qual Life Outcomes. 2016 Sep 15;14:130. doi: 10.1186/s12955-016-0533-4 (PMC5024427; doi:10.1186/s12955-016-0533-4)
Supplement: Additional file 1: — Demographic characteristics by response month. (PDF 126 kb) [file 12955_2016_533_MOESM1_ESM.pdf]

Additional file 1: Demographic characteristics by response month[a]

Characteristic	July (n = 599)	October (n = 4674)	p-values[b]	Total (n = 5273)
Age (mean, sd)	50.3 (17.5)	48.6 (18.0)	0.031	48.8 (17.9)
Gender (%)			0.211	
- female	56.3	53.6		53.9
- male	43.7	46.4		46.1
Ethnicity (%)			0.309	
- native Dutch	85.3	83.1		83.3
- foreign, Western country	7.2	7.0		7.0
- foreign, Non-Western country	3.8	4.9		4.8
- unknown	3.7	5.0		4.9
Education (%)			0.537	
- primary	10.0	9.5		9.5
- lower vocational	21.5	24.3		24.0
- secondary	13.7	11.6		11.8
- middle vocational	23.8	23.0		23.1
- higher vocational	22.8	22.4		22.4
- university	8.0	9.0		8.9
- unknown	0.2	0.3		0.3
Marital status (%)			0.107	
- married	58.4	54.3		54.8
- divorced	9.7	9.1		9.1
- widowed	5.2	5.0		5.0
- never married	26.7	31.7		31.1
Employment status (%)			0.180	
- paid work	50.8	50.7		50.7
- unemployed	3.7	3.4		3.4
- disabled	3.5	3.9		3.8
- school or study	8.7	11.1		10.8
- retired	18.7	19.6		19.5
- household	10.4	7.8		8.1
- other	4.3	3.5		3.6
Monthly net income (%)			0.577	
- 0–500 Euro	19.2	18.1		18.2
- 501–1500 Euro	35.9	35.0		35.1
- 1501–2500 Euro	30.9	31.2		31.2
- > 2500 Euro	9.8	9.9		9.9
- unknown	4.2	5.8		5.6

[a] weighted analyses

[b] t-test in case of continuous variables; Chi-square test in case of categorical variables
