# Supplementary material for: The Four-Dimensional Symptom Questionnaire (4DSQ) in the general population: scale structure, reliability, measurement invariance and normative data: a cross-sectional survey
Source: Health Qual Life Outcomes. 2016 Sep 15;14:130. doi: 10.1186/s12955-016-0533-4 (PMC5024427; doi:10.1186/s12955-016-0533-4)
Supplement: Additional file 2: — Demographic characteristics by data set. (PDF 126 kb) [file 12955_2016_533_MOESM2_ESM.pdf]

Additional file 2: Demographic characteristics by data set[a]

Characteristic	Training set (n = 2635)	Validation set (n = 2638)	p-values[b]	Total (n = 5273)
Age (mean, sd)	48.8 (17.8)	48.9 (18.0)	0.842	48.8 (17.9)
Gender (%)			0.559	
- female	54.3	53.5		53.9
- male	45.7	46.5		46.1
Ethnicity (%)			0.983	
- native Dutch	83.5	83.2		83.3
- foreign, Western country	6.9	7.1		7.0
- foreign, Non-Western country	4.9	4.7		4.8
- unknown	4.8	4.9		4.9
Education (%)			0.338	
- primary	8.9	10.2		9.5
- lower vocational	23.8	24.2		24.0
- secondary	12.0	11.6		11.8
- middle vocational	24.2	21.9		23.1
- higher vocational	22.3	22.5		22.4
- university	8.4	9.4		8.9
- unknown	0.3	0.3		0.3
Marital status (%)			0.156	
- married	54.8	54.8		54.8
- divorced	10.0	8.3		9.1
- widowed	4.8	5.2		5.0
- never married	30.4	31.7		31.1
Employment status (%)			0.264	
- paid work	51.7	49.8		50.7
- unemployed	3.7	3.1		3.4
- disabled	3.7	4.0		3.8
- school or study	11.0	10.7		10.8
- retired	19.2	19.9		19.5
- household	7.2	8.9		8.1
- other	3.6	3.6		3.6
Monthly net income (%)			0.199	
- 0–500 Euro	18.1	18.3		18.2
- 501–1500 Euro	35.9	34.4		35.1
- 1501–2500 Euro	31.3	31.0		31.2
- > 2500 Euro	9.0	10.9		10.0
- unknown	5.7	5.4		5.6

[a] weighted analyses

[b] t-test in case of continuous variables; Chi-square test in case of categorical variables
